# 2-[4-(2-Hy­droxy­propan-2-yl)-1*H*-1,2,3-triazol-1-yl]phenol

**DOI:** 10.1107/S1600536812012925

**Published:** 2012-03-31

**Authors:** Li-Li Zhang, Kai Yu, Ling-Ling Liu, Dian-Shun Guo

**Affiliations:** a Department of Chemistry, Shandong Normal University, Jinan 250014, People’s Republic of China

## Abstract

In the title compound, C_11_H_13_N_3_O_2_, the 1,2,3-triazole ring and the phenol ring form a dihedral angle of 55.46 (5)°. In the crystal, inversion-related mol­ecules associate through pairs of hy­droxy–phenol O—H⋯O hydrogen bonds, giving centrosymmetric cyclic dimers [graph set *R*_2_^2^(18)]. These dimers are linked into infinite chains along [001], giving an overall two-dimensional network structure parallel to the *bc* plane through hy­droxy O—H⋯N and triazole C—H⋯N hydrogen bonds.

## Related literature

For general background to 1,2,3-triazole derivatives, see: Shia *et al.* (2002 ▶); Orgueira *et al.* (2005 ▶); Crowley & Bandeen (2010 ▶). For related structures, see: Zou *et al.* (2006 ▶); Danielraj *et al.* (2010 ▶); Stöger *et al.* (2011 ▶). For bond-length data, see: Banerjee *et al.* (2002 ▶); Janas & Sobota (2005 ▶). For hydrogen-bond motifs, see: Bernstein *et al.* (1995 ▶).
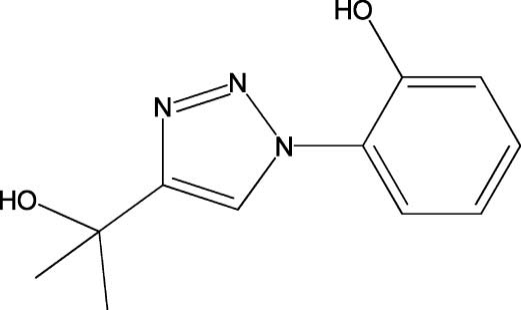


## Experimental

### 

#### Crystal data

C_11_H_13_N_3_O_2_
*M* *_r_* = 219.24
Monoclinic, 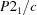

*a* = 11.599 (2) Å
*b* = 9.0747 (18) Å
*c* = 10.743 (2) Å
β = 107.081 (3)°
*V* = 1080.9 (3) Å^3^
*Z* = 4
Mo *K*α radiation
μ = 0.10 mm^−1^
*T* = 298 K
0.40 × 0.30 × 0.18 mm

#### Data collection

Bruker SMART CCD area-detector diffractometer
Absorption correction: multi-scan (*SADABS*; Bruker, 1999 ▶) *T*_min_ = 0.963, *T*_max_ = 0.983
5462 measured reflections
1994 independent reflections
1696 reflections with *I* > 2σ(*I*)
*R*_int_ = 0.025

#### Refinement

*R*[*F* ^2^ > 2σ(*F* ^2^)] = 0.041
*wR*(*F* ^2^) = 0.109
*S* = 1.05
1994 reflections
149 parameters
H-atom parameters constrained
Δρ_max_ = 0.19 e Å^−3^
Δρ_min_ = −0.24 e Å^−3^

### 

Data collection: *SMART* (Bruker, 1999 ▶); cell refinement: *SAINT* (Bruker, 1999 ▶); data reduction: *SAINT*; program(s) used to solve structure: *SHELXS97* (Sheldrick, 2008 ▶); program(s) used to refine structure: *SHELXL97* (Sheldrick, 2008 ▶); molecular graphics: *SHELXTL* (Sheldrick, 2008 ▶); software used to prepare material for publication: *SHELXTL*.

## Supplementary Material

Crystal structure: contains datablock(s) I, global. DOI: 10.1107/S1600536812012925/zs2192sup1.cif

Structure factors: contains datablock(s) I. DOI: 10.1107/S1600536812012925/zs2192Isup2.hkl

Supplementary material file. DOI: 10.1107/S1600536812012925/zs2192Isup3.cml

Additional supplementary materials:  crystallographic information; 3D view; checkCIF report

## Figures and Tables

**Table 1:** Hydrogen-bond geometry (Å, °)

*D*—H⋯*A*	*D*—H	H⋯*A*	*D*⋯*A*	*D*—H⋯*A*
O2—H2⋯O1^i^	0.82	1.89	2.7090 (15)	173
O1—H1⋯N3^ii^	0.82	2.05	2.8665 (16)	171
C7—H7⋯N2^ii^	0.93	2.40	3.2738 (19)	157

Symmetry codes: (i) 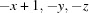; (ii) 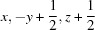.

**Fig. 1.**
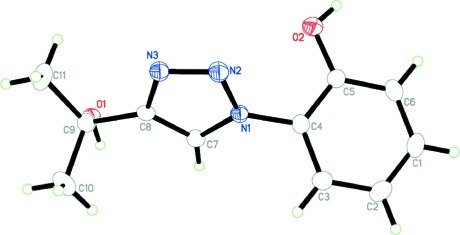
The molecular structure of the title compound with displacement ellipsoids drawn at the the 30% probability level.

**Fig. 2.**
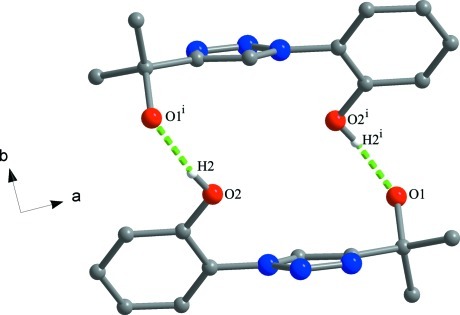
A centrosymmetric dimer of the title compound formed by intermolecular O—H···O hydrogen bonds, showing the R22(18) motif. For the sake of clarity, H atoms not involved in the motif have been omitted. For symmetry code (i), see Table 1.

**Fig. 3.**
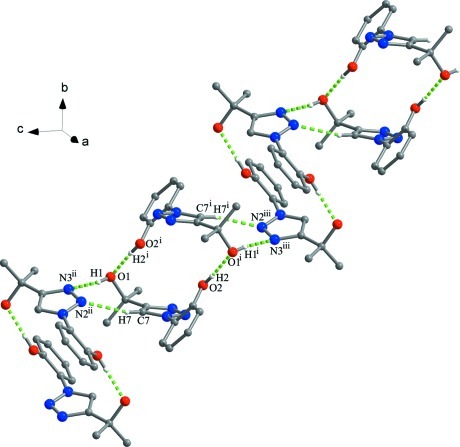
A hydrogen-bonded chain of the title compound, showing the R22(18) and R22(8) motifs. For the sake of clarity, H atoms not involved in the motifs have been omitted. For symmetry code (iii), -x + 1, y - 1/2, -z - 1/2. For symmetry code (ii), see Table 1.

## supplementary crystallographic information

### Comment

1,2,3-Triazole derivatives have received much attention owing to their wide applications in drug discovery, materials and supramolecular chemistry (Shia *et al.*, 2002; Orgueira *et al.*, 2005; Crowley & Bandeen 2010). Numerous crystal structures of triazole derivatives have been described (Danielraj *et al.*, 2010; Stöger *et al.*, 2011). We report here the structure of a new triazole compound, 2-[4-(2-hydroxypropan-2-yl)-1*H*-1,2,3-triazol-1-yl]phenol, C_11_H_13_N_3_O_2_.

The title compound, shown in Fig. 1, contains a 1,2,3-triazole ring and a phenol ring which are non-coplanar with a dihedral angle of 55.46 (5)°, larger than that reported previously for a similar structure [14.34 (17)°] (Zou *et al.*, 2006). This difference may be ascribed to the steric repulsion between the heterocyclic N atom, N2, and the phenolic hydroxyl oxygen atom, O2. The bond lengths of C7—N1, C8—N3 and N1—N2 are shorter than the normal C—N single bond length (1.483 Å) (Banerjee *et al.*, 2002) and N—N single bond length (1.467 Å) (Janas & Sobota, 2005), showing an obvious electron delocalization in the triazole ring.

The packing of the title compound is stabilized by intermolecular O—H···O, O—H···N and C—H···N hydrogen bonds (Table 1). Two inversion- related molecules form a centrosymmetric dimer through intermolecular hydroxyl O2—H···O1^i^ hydrogen bonds, locally creating an *R*^2^_2_(18) motif (Bernstein *et al.*, 1995) (Fig. 2). These dimers are linked into chains which give an overall two-dimensional network structure through intermolecular hydroxyl O1—H···N3^ii^ and triazole C7—H7···N2^ii^ hydrogen-bonding interactions, which include a cyclic *R*^2^_2_(8) motif (Fig. 3). For symmetry codes (i) and (ii), see Table 1.

### Experimental

2-Methylbut-3-yn-2-ol (0.093 g, 1.1 mmol) was added to a suspension of 2-azidophenol (0.135 g, 1.0 mmol), CuI (0.019 g, 0.10 mmol), Et_3_N (0.5 ml) and ascorbic acid (0.018 g, 0.10 mmol) in CH_3_CN (2.0 ml) and continuously stirred at 298 K for 0.5 h. The resulting mixture was extracted with CH_2_Cl_2_ and the organic layer was washed with brine, then dried over anhydrous MgSO_4_. After removal of the solvent under reduced pressure, the crude product was purified by recrystallization from CH_2_Cl_2_/pentane to afford the title compound as a pale yellow solid (95% yield). Single crystals of the title compound suitable for X-ray diffraction analysis were obtained by slow diffusion of pentane into a solution of the title compound in CH_2_Cl_2_ at 298 K.

### Refinement

All H atoms were placed in geometrically idealized positions and refined using a riding model with C—H = 0.93 Å and *U*_iso_(H)= 1.2*U*_eq_(C) (aromatic); C—H = 0.96 Å and *U*_iso_(H) = 1.5*U*_eq_(C) (methyl); O—H= 0.82 Å and *U*_iso_(H)= 1.5*U*_eq_(O) (hydroxyl).

### Crystal data

**Table:**

C_11_H_13_N_3_O_2_	*F*(000) = 464
*M**_r_* = 219.24	*D*_x_ = 1.347 Mg m^−^^3^
Monoclinic, *P*2_1_/*c*	Mo *K*α radiation, λ = 0.71073 Å
Hall symbol: -P 2ybc	Cell parameters from 2071 reflections
*a* = 11.599 (2) Å	θ = 2.9–23.0°
*b* = 9.0747 (18) Å	µ = 0.10 mm^−^^1^
*c* = 10.743 (2) Å	*T* = 298 K
β = 107.081 (3)°	Block, pale yellow
*V* = 1080.9 (3) Å^3^	0.40 × 0.30 × 0.18 mm
*Z* = 4	

### Data collection

**Table:**

Bruker SMART CCD area-detector diffractometer	1994 independent reflections
Radiation source: fine-focus sealed tube	1696 reflections with *I* > 2σ(*I*)
Graphite monochromator	*R*_int_ = 0.025
φ and ω scans	θ_max_ = 25.5°, θ_min_ = 1.8°
Absorption correction: multi-scan (*SADABS*; Bruker, 1999)	*h* = −14→12
*T*_min_ = 0.963, *T*_max_ = 0.983	*k* = −10→10
5462 measured reflections	*l* = −11→13

### Refinement

**Table:**

Refinement on *F*^2^	Primary atom site location: structure-invariant direct methods
Least-squares matrix: full	Secondary atom site location: difference Fourier map
*R*[*F*^2^ > 2σ(*F*^2^)] = 0.041	Hydrogen site location: inferred from neighbouring sites
*wR*(*F*^2^) = 0.109	H-atom parameters constrained
*S* = 1.05	*w* = 1/[σ^2^(*F*_o_^2^) + (0.0589*P*)^2^ + 0.1715*P*] where *P* = (*F*_o_^2^ + 2*F*_c_^2^)/3
1994 reflections	(Δ/σ)_max_ < 0.001
149 parameters	Δρ_max_ = 0.19 e Å^−^^3^
0 restraints	Δρ_min_ = −0.24 e Å^−^^3^

### Special details

**Table:**

Geometry. All e.s.d.'s (except the e.s.d. in the dihedral angle between two l.s. planes) are estimated using the full covariance matrix. The cell e.s.d.'s are taken into account individually in the estimation of e.s.d.'s in distances, angles and torsion angles; correlations between e.s.d.'s in cell parameters are only used when they are defined by crystal symmetry. An approximate (isotropic) treatment of cell e.s.d.'s is used for estimating e.s.d.'s involving l.s. planes.
Refinement. Refinement of *F*^2^ against ALL reflections. The weighted *R*-factor *wR* and goodness of fit *S* are based on *F*^2^, conventional *R*-factors *R* are based on *F*, with *F* set to zero for negative *F*^2^. The threshold expression of *F*^2^ > σ(*F*^2^) is used only for calculating *R*-factors(gt) *etc*. and is not relevant to the choice of reflections for refinement. *R*-factors based on *F*^2^ are statistically about twice as large as those based on *F*, and *R*- factors based on ALL data will be even larger.

### Fractional atomic coordinates and isotropic or equivalent isotropic displacement parameters (Å^2^)

**Table:**

	*x*	*y*	*z*	*U*_iso_*/*U*_eq_	
N1	0.57130 (10)	0.27595 (13)	0.05416 (11)	0.0285 (3)	
N2	0.50492 (12)	0.30872 (16)	−0.06770 (11)	0.0374 (3)	
N3	0.39371 (11)	0.32767 (15)	−0.06404 (11)	0.0352 (3)	
O1	0.26044 (9)	0.18448 (11)	0.16529 (9)	0.0321 (3)	
H1	0.2976	0.1914	0.2428	0.048*	
O2	0.65112 (10)	0.05481 (13)	−0.07335 (11)	0.0447 (3)	
H2	0.6832	−0.0144	−0.0993	0.067*	
C1	0.93917 (15)	0.1799 (2)	0.14569 (17)	0.0443 (4)	
H1A	1.0210	0.1584	0.1663	0.053*	
C2	0.90012 (16)	0.2893 (2)	0.21198 (17)	0.0478 (5)	
H2A	0.9552	0.3409	0.2780	0.057*	
C3	0.77857 (14)	0.32227 (18)	0.18018 (15)	0.0391 (4)	
H3	0.7517	0.3971	0.2239	0.047*	
C4	0.69727 (13)	0.24373 (16)	0.08337 (13)	0.0293 (3)	
C5	0.73520 (13)	0.13098 (17)	0.01699 (14)	0.0314 (4)	
C6	0.85794 (14)	0.10189 (19)	0.04891 (15)	0.0389 (4)	
H6	0.8857	0.0287	0.0043	0.047*	
C7	0.50258 (13)	0.27627 (17)	0.13542 (13)	0.0312 (4)	
H7	0.5273	0.2581	0.2245	0.037*	
C8	0.38921 (13)	0.30876 (15)	0.05978 (13)	0.0276 (3)	
C9	0.27444 (13)	0.32016 (16)	0.09872 (14)	0.0312 (4)	
C10	0.28121 (17)	0.44984 (19)	0.18952 (18)	0.0495 (5)	
H10A	0.2095	0.4530	0.2167	0.074*	
H10B	0.2881	0.5396	0.1449	0.074*	
H10C	0.3503	0.4391	0.2645	0.074*	
C11	0.16508 (15)	0.3294 (2)	−0.01930 (17)	0.0495 (5)	
H11A	0.1618	0.2438	−0.0727	0.074*	
H11B	0.1704	0.4162	−0.0684	0.074*	
H11C	0.0935	0.3341	0.0082	0.074*	

### Atomic displacement parameters (Å^2^)

**Table:**

	*U*^11^	*U*^22^	*U*^33^	*U*^12^	*U*^13^	*U*^23^
N1	0.0282 (6)	0.0342 (7)	0.0230 (6)	0.0021 (5)	0.0074 (5)	0.0005 (5)
N2	0.0356 (7)	0.0538 (9)	0.0229 (6)	0.0069 (6)	0.0088 (5)	0.0035 (6)
N3	0.0327 (7)	0.0475 (8)	0.0254 (6)	0.0055 (6)	0.0083 (5)	0.0012 (6)
O1	0.0347 (6)	0.0357 (6)	0.0252 (5)	−0.0038 (4)	0.0077 (4)	−0.0015 (4)
O2	0.0401 (7)	0.0444 (7)	0.0469 (7)	0.0023 (5)	0.0084 (5)	−0.0152 (5)
C1	0.0284 (8)	0.0522 (11)	0.0519 (10)	0.0023 (7)	0.0114 (8)	0.0061 (8)
C2	0.0364 (10)	0.0517 (11)	0.0479 (10)	−0.0050 (8)	0.0010 (8)	−0.0062 (8)
C3	0.0380 (9)	0.0414 (9)	0.0358 (9)	0.0011 (7)	0.0079 (7)	−0.0057 (7)
C4	0.0293 (8)	0.0325 (8)	0.0276 (7)	0.0014 (6)	0.0106 (6)	0.0045 (6)
C5	0.0317 (8)	0.0341 (8)	0.0291 (8)	−0.0019 (6)	0.0100 (6)	0.0018 (6)
C6	0.0376 (9)	0.0413 (9)	0.0422 (9)	0.0056 (7)	0.0187 (7)	0.0014 (7)
C7	0.0326 (8)	0.0404 (9)	0.0218 (7)	0.0003 (6)	0.0098 (6)	0.0011 (6)
C8	0.0324 (8)	0.0276 (8)	0.0224 (7)	−0.0006 (6)	0.0077 (6)	−0.0024 (6)
C9	0.0309 (8)	0.0322 (8)	0.0319 (8)	0.0018 (6)	0.0113 (6)	0.0025 (6)
C10	0.0594 (12)	0.0372 (10)	0.0632 (12)	0.0013 (8)	0.0355 (10)	−0.0056 (8)
C11	0.0327 (9)	0.0685 (13)	0.0466 (10)	0.0057 (8)	0.0107 (8)	0.0180 (9)

### Geometric parameters (Å, º)

**Table:**

N1—N2	1.3428 (16)	C3—H3	0.9300
N1—C7	1.3441 (18)	C4—C5	1.390 (2)
N1—C4	1.4316 (19)	C5—C6	1.388 (2)
N2—N3	1.3134 (18)	C6—H6	0.9300
N3—C8	1.3575 (18)	C7—C8	1.360 (2)
O1—C9	1.4568 (18)	C7—H7	0.9300
O1—H1	0.8200	C8—C9	1.512 (2)
O2—C5	1.3472 (18)	C9—C11	1.510 (2)
O2—H2	0.8200	C9—C10	1.516 (2)
C1—C2	1.374 (3)	C10—H10A	0.9600
C1—C6	1.376 (2)	C10—H10B	0.9600
C1—H1A	0.9300	C10—H10C	0.9600
C2—C3	1.383 (2)	C11—H11A	0.9600
C2—H2A	0.9300	C11—H11B	0.9600
C3—C4	1.379 (2)	C11—H11C	0.9600
			
N2—N1—C7	110.68 (12)	N1—C7—C8	105.44 (12)
N2—N1—C4	121.00 (11)	N1—C7—H7	127.3
C7—N1—C4	128.31 (12)	C8—C7—H7	127.3
N3—N2—N1	106.67 (11)	N3—C8—C7	107.72 (13)
N2—N3—C8	109.48 (12)	N3—C8—C9	123.57 (13)
C9—O1—H1	109.5	C7—C8—C9	128.70 (13)
C5—O2—H2	109.5	O1—C9—C11	105.78 (12)
C2—C1—C6	120.43 (15)	O1—C9—C8	108.19 (11)
C2—C1—H1A	119.8	C11—C9—C8	111.25 (12)
C6—C1—H1A	119.8	O1—C9—C10	109.42 (12)
C1—C2—C3	119.74 (16)	C11—C9—C10	111.68 (14)
C1—C2—H2A	120.1	C8—C9—C10	110.36 (13)
C3—C2—H2A	120.1	C9—C10—H10A	109.5
C4—C3—C2	119.74 (15)	C9—C10—H10B	109.5
C4—C3—H3	120.1	H10A—C10—H10B	109.5
C2—C3—H3	120.1	C9—C10—H10C	109.5
C3—C4—C5	121.17 (14)	H10A—C10—H10C	109.5
C3—C4—N1	119.17 (13)	H10B—C10—H10C	109.5
C5—C4—N1	119.62 (13)	C9—C11—H11A	109.5
O2—C5—C6	123.59 (14)	C9—C11—H11B	109.5
O2—C5—C4	118.42 (13)	H11A—C11—H11B	109.5
C6—C5—C4	117.99 (14)	C9—C11—H11C	109.5
C1—C6—C5	120.90 (15)	H11A—C11—H11C	109.5
C1—C6—H6	119.5	H11B—C11—H11C	109.5
C5—C6—H6	119.5		
			
C7—N1—N2—N3	−0.92 (17)	C2—C1—C6—C5	−0.6 (3)
C4—N1—N2—N3	177.94 (12)	O2—C5—C6—C1	−177.47 (15)
N1—N2—N3—C8	0.68 (16)	C4—C5—C6—C1	1.6 (2)
C6—C1—C2—C3	−0.7 (3)	N2—N1—C7—C8	0.77 (16)
C1—C2—C3—C4	0.9 (3)	C4—N1—C7—C8	−177.98 (14)
C2—C3—C4—C5	0.2 (2)	N2—N3—C8—C7	−0.22 (17)
C2—C3—C4—N1	178.08 (14)	N2—N3—C8—C9	−178.97 (13)
N2—N1—C4—C3	125.90 (15)	N1—C7—C8—N3	−0.34 (16)
C7—N1—C4—C3	−55.5 (2)	N1—C7—C8—C9	178.33 (14)
N2—N1—C4—C5	−56.22 (19)	N3—C8—C9—O1	125.29 (14)
C7—N1—C4—C5	122.42 (16)	C7—C8—C9—O1	−53.19 (19)
C3—C4—C5—O2	177.68 (14)	N3—C8—C9—C11	9.5 (2)
N1—C4—C5—O2	−0.2 (2)	C7—C8—C9—C11	−168.97 (16)
C3—C4—C5—C6	−1.5 (2)	N3—C8—C9—C10	−115.03 (16)
N1—C4—C5—C6	−179.33 (13)	C7—C8—C9—C10	66.5 (2)

### Hydrogen-bond geometry (Å, º)

**Table:**

*D*—H···*A*	*D*—H	H···*A*	*D*···*A*	*D*—H···*A*
O2—H2···O1^i^	0.82	1.89	2.7090 (15)	173
O1—H1···N3^ii^	0.82	2.05	2.8665 (16)	171
C7—H7···N2^ii^	0.93	2.40	3.2738 (19)	157

Symmetry codes: (i) −*x*+1, −*y*, −*z*; (ii) *x*, −*y*+1/2, *z*+1/2.
